# Effects of SGLT2 inhibitor dapagliflozin in patients with type 2 diabetes on skeletal muscle cellular metabolism

**DOI:** 10.1016/j.molmet.2022.101620

**Published:** 2022-10-21

**Authors:** Yvo J.M. op den Kamp, Anne Gemmink, Marlies de Ligt, Bas Dautzenberg, Esther Kornips, Johanna A. Jorgensen, Gert Schaart, Russell Esterline, Diego A. Pava, Joris Hoeks, Vera B. Schrauwen-Hinderling, Sander Kersten, Bas Havekes, Timothy R. Koves, Deborah M. Muoio, Matthijs K.C. Hesselink, Jan Oscarsson, Esther Phielix, Patrick Schrauwen

**Affiliations:** 1 Departments of Nutrition and Movement Sciences, Maastricht, the Netherlands; 2 Departments of Radiology and Nuclear Medicine, Maastricht, the Netherlands; 3 Departments of Internal Medicine, Division of Endocrinology, NUTRIM School of Nutrition and Translational Research in Metabolism, Maastricht University Medical Center, 6200 MD Maastricht, the Netherlands; 4 BioPharmaceuticals R&D, AstraZeneca, Gaithersburg, MD, USA; 5 Duke Molecular Physiology Institute and the Sarah W. Stedman Nutrition and Metabolism Center, Department of Medicine, Duke University, Durham, NC 27704, USA; 6 Division of Human Nutrition and Health, Wageningen University, the Netherlands; 7 BioPharmaceuticals R&D, Late-Stage Development, Cardiovascular, Renal and Metabolism, AstraZeneca, Gothenburg, Sweden

**Keywords:** Acylcarnitines, Dapagliflozin, Myocellular lipid metabolism, Mitochondrial function, SGLT2i, TCA cycle Intermediates

## Abstract

**Objective:**

SGLT2 inhibitors increase urinary glucose excretion and have beneficial effects on cardiovascular and renal outcomes; the underlying mechanism may be metabolic adaptations due to urinary glucose loss. Here, we investigated the cellular and molecular effects of 5 weeks of dapagliflozin treatment on skeletal muscle metabolism in type 2 diabetes patients.

**Methods:**

Twenty-six type 2 diabetes mellitus patients were randomized to a 5-week double-blind, cross-over study with 6-8-week wash-out. Skeletal muscle acetylcarnitine levels, intramyocellular lipid (IMCL) content and phosphocreatine (PCr) recovery rate were measured by magnetic resonance spectroscopy (MRS). *Ex vivo* mitochondrial respiration was measured in skeletal muscle fibers using high resolution respirometry. Intramyocellular lipid droplet and mitochondrial network dynamics were investigated using confocal microscopy. Skeletal muscle levels of acylcarnitines, amino acids and TCA cycle intermediates were measured. Expression of genes involved in fatty acid metabolism were investigated.

**Results:**

Mitochondrial function, mitochondrial network integrity and citrate synthase and carnitine acetyltransferase activities in skeletal muscle were unaltered after dapagliflozin treatment. Dapagliflozin treatment increased intramyocellular lipid content (0.060 (0.011, 0.110) %, p = 0.019). Myocellular lipid droplets increased in size (0.03 μm^2^ (0.01–0.06), p < 0.05) and number (0.003 μm^−2^ (−0.001–0.007), p = 0.09) upon dapagliflozin treatment. CPT1A, CPT1B and malonyl CoA-decarboxylase mRNA expression was increased by dapagliflozin. Fasting acylcarnitine species and C4–OH carnitine levels (0.4704 (0.1246, 0.8162) pmoles∗mg tissue^−1^, p < 0.001) in skeletal muscle were higher after dapagliflozin treatment, while acetylcarnitine levels were lower (−40.0774 (−64.4766, −15.6782) pmoles∗mg tissue^−1^, p < 0.001). Fasting levels of several amino acids, succinate, alpha-ketoglutarate and lactate in skeletal muscle were significantly lower after dapagliflozin treatment.

**Conclusion:**

Dapagliflozin treatment for 5 weeks leads to adaptive changes in skeletal muscle substrate metabolism favoring metabolism of fatty acid and ketone bodies and reduced glycolytic flux.

The trial is registered with ClinicalTrials.gov, number NCT03338855.

## Highlights

• Dapagliflozin treatment for 5 weeks increases intramyocellular lipid content in type 2 diabetes patients.
• Change in intramyocellular lipid droplet dynamics mainly resemble changes after caloric restriction.
• Dapagliflozin increased gene expression levels reflecting increased lipid metabolism.
• Metabolomics in skeletal muscle reveals increased fatty acid use for energy production after 5 weeks of dapagliflozin.
• No change in skeletal muscle mitochondrial function was observed after 5 weeks of dapagliflozin treatment.

## Graphical abstract

**Figure:**
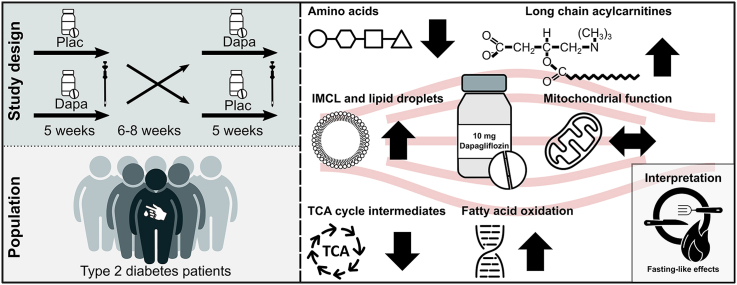

## Abbreviations

α-Kg: α-ketoglutarate
ACAC: Acetyl-CoA carboxylase
CV: Cardiovascular
CrAT: Carnitine acetyltransferase
CPT: Carnitine palmitoyltransferase
DGAT: Diglyceride acyltranferase
FASN: Fatty acid synthase
FFA: Free fatty acids
IHL: Intrahepatic lipid
IMCL: Intramyocellular lipid
IMF: Intermyofibrillar
LD: Lipid droplet
MFI: Mitochondrial fragmentation index
MLYCD: Malonyl-CoA decarboxylase
MRS: Magnetic resonance spectroscopy
MRUM: Metabolic Research Unit Maastricht
PCr: Phosphocreatine
SCD: Stearoyl-CoaA desaturase
SGLT2i: Sodium-glucose cotransporter 2 inhibitors
SS: Subsarcolemmal
TCA cycle: Tricarboxylic acid cycle

## 1 Introduction

Sodium-glucose cotransporter 2 inhibitors (SGLT2i) reduce renal glucose reabsorption in the proximal tubules and increase urinary glucose excretion. The treatment was initially developed to improve glycemic control in patients with type 2 diabetes, but has been shown to have organ protective effects including reduced risk for cardiovascular (CV) events, especially reduced risk for hospitalization for heart failure, and reduced risk for progression of chronic kidney disease [1,2]. The primary action of SGLT2i on glucose reabsorption has several metabolic consequences that may help to explain the effects on CV and renal outcome [3]. Such metabolic effects may be the consequence of the adaptive response to the loss of about 50–100 g glucose per day in the urine, which can be regarded as a form of mild calorie restriction. Since SGLT2 is almost exclusively expressed in the kidney [4], the metabolic effects of SGLT2 inhibitors on skeletal muscle and other tissues are indirect effects and most likely explained by the urinary glucose loss induced by these inhibitors. Moreover, *in vitro* studies using cells that do not express SGLT2 and using therapeutic doses of dapagliflozin did not show any effects on cellular metabolism [5]. Such urinary glucose loss is also a direct loss of calories, resulting in fasting-like/calorie restriction-like effects [6], such as an increased plasma free fatty acids and fat oxidation, effects that are not observed with other glucose lowering, antidiabetic drugs. Indeed we recently showed that 5 weeks of SGLT2i treatment in type 2 diabetes patients resulted in ∼90 g of glucose excretion via urine, reduced 24 h glucose levels and increased circulating free fatty acids (FFA) and beta-hydroxybutyrate levels, accompanied by marked adjustments of 24 h energy metabolism that (in part) mimic the effects of calorie restriction, such as increased 24 h fat oxidation, improved metabolic flexibility, and hepatic and adipose tissue insulin sensitivity, while whole-body, and peripheral insulin sensitivity was not affected [6]. These findings were in line with and extended previous reports of increased fat oxidation [7], decreased intrahepatic lipid content [8], decreased total body fat mass [3,9], and decreased visceral adipose tissue [9] after SGLT2i treatment of type 2 diabetes patients.

Calorie restriction in humans is accompanied by adaptations in skeletal muscle metabolism, which can help to explain the underlying metabolic health effects of such interventions. Thus, calorie restriction has been shown to improve skeletal muscle fat oxidative and mitochondrial capacity [10]. Such adaptations are important, as we have previously shown that high whole-body and mitochondrial fatty acid oxidation capacity attenuates lipotoxicity [11]. Furthermore, lipid-induced insulin resistance in skeletal muscle is associated with reduced mitochondrial function [12,13] and increased mitochondrial network fragmentation [14]. Indeed, under diabetogenic conditions, lipid supply to skeletal muscle may exceed mitochondrial oxidative capacity, resulting in the accumulation of intramyocellular lipids (IMCL) and accompanying insulin resistance. It has been suggested that carnitine acetyltransferase (CrAT) can function as a defense mechanism against such mitochondrial substrate oversupply. Thus, excessive mitochondrial acetyl-CoA can be converted by CrAT to acetylcarnitine [15] and thereby reduce the allosteric inhibition on the pyruvate dehydrogenase complex and subsequently increase mitochondrial glucose oxidation [16]. Alleviating substrate competition at the level of mitochondria [17], could improve metabolic flexibility in patients with type 2 diabetes. Indeed, the capacity to form acetylcarnitine in skeletal muscle has been suggested to be a determinant of insulin sensitivity [18,19]. Another putative defense mechanism against mitochondrial substrate oversupply and thereby the prevention of insulin resistance is lipid droplet (LD) remodeling [20]. Under diabetic conditions, high IMCL content is a consequence of large LDs. These LDs are mainly found in the subsarcolemmal region [21]. We [22] and others [23] have shown that the ability to remodel the LD pool, i.e. the ability to change LD morphology and protein coating of LDs upon a high fatty acid influx, is associated with maintained insulin sensitivity.

Preclinical studies have shown that SGLT2i restored [24] or improved [25] mitochondrial function in heart failure. Therefore, to further test the hypothesis that SGLT2 inhibition induces calorie restriction-like effects, we here examined if SGLT2 inhibition exerts calorie restriction-like effects on skeletal muscle metabolism, such as improved mitochondrial function and fatty acid metabolism at the skeletal muscle level as observed with calorie restriction [10]. This is also relevant as improvements in myocellular fatty acid metabolism may be early adaptations underlying other secondary effects, such as improved peripheral insulin sensitivity observed by others upon SGLT2i treatment [26,27]. To this end, we investigated the indirect effect of 5 weeks of SGLT2i treatment on skeletal muscle substrate handling and aimed to explore the effect of dapagliflozin treatment on mitochondrial function, mitochondrial network integrity, mitochondrial substrate competition, LD remodeling, and the capacity to form acetylcarnitine.

## 2 Materials and methods

### 2.1 Study design and participants

A double-blind, randomized, placebo-controlled, cross-over Phase IV trial study, was conducted at the Metabolic Research Unit Maastricht (MRUM) of Maastricht University as previously reported [6]. The study took place between 5 March 2018 and 4 November 2019. The study protocol was approved by the Ethics Committee of Maastricht University Medical Center and was conducted conform to the declaration of Helsinki [28]. Patients were randomized to a double-blind, placebo-controlled intervention study with 2 treatment periods, each of 5 weeks or a maximum duration of 40 days, separated by a wash-out period of 6–8 weeks. Endpoints were assessed at the end of each 5-week period. In brief, the target population consisted of patients with type 2 diabetes diagnosed for at least 6 months who had been stable on a dose of metformin and/or a DPPIV inhibitor for the previous 3 months or more or were drug naïve. Patients were to have HbA1c levels between 6% and 9% (42 and 75 mmol/mol). A table with all inclusion and exclusion criteria was previously published [6]. Written informed consent was obtained from all participants before inclusion. Detailed descriptions of the procedures, not described below, can be found in the supplemental materials.

### 2.2 Magnetic resonance spectroscopy measurements

On the first day of Visit 4 and 7, at 3:00 P.M., *in vivo* IMCL content in tibialis anterior muscle was assessed by ^1^H-MRS on a 3.0 T whole-body magnetic resonance system (Achieva 3Tx; Philips Healthcare) as described previously [29]. Subsequently, *in vivo* mitochondrial oxidative capacity was determined by ^31^P-MRS at 4:00 P.M., as previously described [30]. On a separate day, at the last day of the end-of-treatment visits (to prevent effects of exercise testing on the other outcome parameters), acetylcarnitine concentrations were acquired by ^1^H-MRS in skeletal muscle before and after exercise. Participants were fasted from 12:00 A.M. and were asked to refrain from strenuous physical activity 72 h before the measurement. Detailed descriptions of the different MRS measurement procedures can be found in the supplemental materials.

### 2.3 Muscle biopsy

On day 3 of the end-of-treatment visit, a percutaneous muscle biopsy was obtained from the vastus lateralis muscle in the fasted state before the start of a hyperinsulinemic euglycemic clamp and as described previously [31], under local anaesthesia (1% lidocaine), as described by Bergström et al. [32]. A small portion of tissue was immediately placed in preservation medium (BIOPS; Oroboros Instruments, Innsbruck, Austria). Muscle fibers were separated with small needles and the muscle membrane was permeabilized with a Saponin stock solution (5 mg/mL BIOPS), as previously described [31]. Saponin was removed and ∼3–4 mg wet weight fiber was transferred into the oxygraph. The remainder of the biopsy was immediately snap frozen and stored at −80 °C, for assessment of carnitine acetyltransferase (CrAT) and citrate synthase (CS) activity, and levels of acylcarnitines, amino acids, TCA cycle intermediates, and mRNA measurements. Additional skeletal muscle tissue was placed in isopentane, and then frozen in liquid nitrogen and stored at −80 °C for further microscopy and biochemical analyses. Two participants did not complete the clinical trial [6], and from one participant there was no muscle biopsy available from the first period. This resulted in a total of 23 participants from who we had a muscle biopsy available from both periods.

### 2.4 High-resolution respirometry

Muscle fibers were permeabilized as previously described [31]. High resolution respirometry was used to measure *ex vivo* mitochondrial respiration, under hyperoxic conditions at 37 °C in a two-chamber oxygraphy (Oroboros, Innsbruck, Austria) and expressed as pmol ∗ mg^−1^ muscle fiber wet weight ∗ s^−1^. Oxidative phosphorylation was measured by adding 4.0 mmol/L malate, 10.0 mmol/L glutamate, 2.0 mmol/L ADP and 10.0 mmol/L succinate, with or without the presence of 40 μmol/L octanoylcarnitine. Leak respiration or maximal respiratory capacity was determined by adding respectively 2.0 μg/mL oligomycin or 0.5 μmol/L titrations of uncoupler fluoro-carbonyl cyanide phenylhydrazone. Cytochrome C (10.0 mmol/L) was added to check the integrity of mitochondrial outer membrane, and revealed good quality of all permeabilized mitochondrial analysis.

### 2.5 Biochemical analysis

Acylcarnitines, amino acids and TCA cycle intermediates were analyzed in skeletal muscle tissue obtained from the muscle biopsy taken visit 4 and 7 by flow injection tandem mass spectrometry, using sample preparation methods described previously [33,34]. Skeletal muscle TCA cycle intermediates were measured using gas chromatography-mass spectrometry as previously described [35]. Data for the analysis of acylcarnitines and amino acids were acquired using a Waters AcquityTM UPLC system with a TQ (triple quadrupole) detector. The data system was controlled by MassLynx 4.1 operating system (Waters, Milford, MA). Plasma levels of lactate (Roche, Basel, Switzerland) was analyzed enzymatically in EDTA samples using a Pentra 400 (Horiba).

### 2.6 Confocal microscopy analyses

#### 2.6.1 Subject selection

The confocal microscopy analyses of lipid droplet morphology were performed in a subset of 10 participants that had an increased IMCL content upon dapagliflozin treatment, based on Bodipy 493/503 (D3922, Molecular Probes, Leiden, The Netherlands) stained muscle biopsy sections using widefield microscopy analyses of IMCL (Supplemental Fig. 1).

#### 2.6.2 Staining procedures, confocal microscopy imaging and image analysis

A detailed description of the staining procedures and the confocal microscopy image analyses can be found in the supplemental materials. For all analyses, i.e. LD morphology and location, mitochondrial network integrity and LD-mitochondrial interaction, 12-bit z-stacks were acquired on a Leica TCS SP8 confocal microscope as previously described [36,37]. Complete z-stacks with a 0.10 μm z-step for LD-mitochondrial interaction were acquired with a 100 × 1.4 N A. oil immersion objective combined with a 5× optical zoom resulting in a 23 by 23 by 100 nm voxel size. All images were deconvolved using Huygens Professional Software (Scientific Volume Imaging B.V., Hilversum, the Netherlands). All images were analyzed in ImageJ [38] with in-home written scripts.

### 2.7 Gene expression for genes related to fatty acid metabolism

To determine expression of genes related to fatty acid metabolism, we used data obtained from RNAseq analysis which was performed for a separate study (manuscript in preparation). In brief, total RNA from all samples (n = 44) was extracted using TRIzol reagent (Thermo Fisher Scientific, the Netherlands) and purified using the Qiagen RNeasy Mini kit (Qiagen, the Netherlands) according to manufacturer's instructions. Library construction and RNA sequencing runs on the BGISEQ-500 platform [39] were conducted at Beijing Genomics Institute (BGI, Denmark). All the generated raw sequencing reads were filtered, by removing reads with adaptors, reads with more than 10% of unknown bases, and low-quality reads. Clean reads were then obtained and stored as FASTQ format. A detailed description of the RNA sequencing and processing these RNA sequencing reads can be found in the supplemental information.

### 2.8 Statistics

The evaluable analysis set, consisting of patients with at least one dose of the investigational product (per protocol) and no important protocol deviations, was used for the statistical analyses, using SPSS version 27 (IBM Corp., Armonk, NY, USA). The expected difference between treatment groups was estimated using a linear mixed effects model. This model had treatment group, treatment sequence and period as fixed effects, as well as random intercept for each subject. This model assumes independent conditional residuals with equal variations in each period and treatment group. Residual plots and tests for normal distribution of model residuals were used to check model assumptions. If deviations from normality were detected, a non-parametric test of treatment difference against zero was performed (Wilcoxon paired signed-rank test) using all the data and ignoring the sequence. The least-squares (LS) means for treatment effect in the respective treatment groups and the corresponding 95% CIs are presented. The difference in LS means between the two treatments was generated, with corresponding 95% CI and p-value tabulated. Pearson correlations were performed using a linear regression model. If deviations from normality was detected, a spearman correlation was performed. A two-sided 0.05 level is considered as statistically significant.

## 3 Results

### 3.1 Participant characteristics

In our previous publication, we have reported the participants characteristics [6]. These characteristics are shown in Table 1. In addition, the effects of 5 weeks of dapagliflozin on glycaemic control have been published before [6] and are shown in Supplemental Table 1.

**Table 1:** Participant characteristics.

Characteristic	Total (n = 24)
Age, years (mean ± SD)	62.4 ± 4.6
Sex, n (male/female)	19/5
BMI, kg/m^2^ (mean ± SD)	28.1 ± 2.4
HbA1c, mmol/mol (mean ± SD)/% (mean ± SD)	51.7 ± 6.8/6.9 ± 0.6
eGFR, ml/min (mean ± SD)	141.0 ± 13.0
Duration of diabetes, years (median (range))	8.0 (1–15)
Metformin use, % (yes/total)	71 (17/24)
Any diabetes complications, n (yes/no)	1/23

### 3.2 Unaltered mitochondrial function and mitochondrial network integrity after dapagliflozin treatment

**Figure 1:**
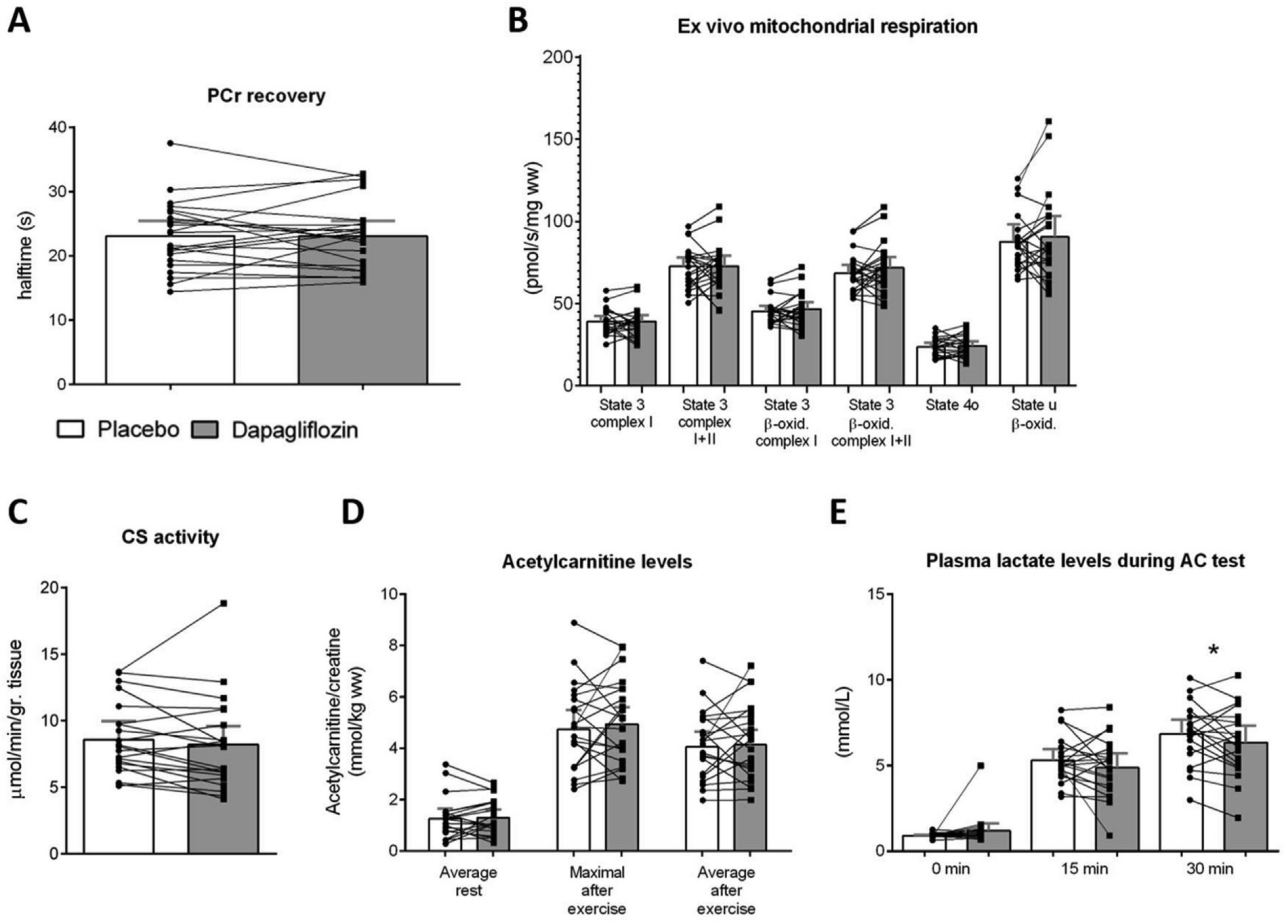
**Effects of dapagliflozin on mitochondrial function and acetylcarnitine levels.(A)** Phosphocreatine (PCr) recovery rate (n = 22), **(B)***ex vivo* mitochondrial respiration from vastus lasteralis muscle biopsies taken after an overnight fast (n = 22), **(C)** Citrate synthase activity (n = 21), **(D)** average acetylcarnitine levels at rest (n = 21), maximal acetylcarnitine levels after exercise (n = 21), and average acetylcarnitine levels after exercise (n = 21), and **(E)** plasma lactate levels during exercise (n = 20) after placebo (P) and dapagliflozin (D) treatment. Placebo condition = white bars, dapagliflozin condition = grey bars. Results are given as least squares mean (LSM) and 95% confidence interval (CI), obtained through a linear mixed model. ∗P < 0.05 vs. placebo by Wilcoxon paired signed-rank test.

We have previously reported that dapagliflozin treatment for 5 weeks increased 24 h whole-body fat oxidation in type 2 diabetes patients [6]. To investigate if these effects were associated with altered mitochondrial function in skeletal muscle, we determined *in vivo* mitochondrial function measured as half-time PCr recovery rate with ^31^P-MRS. *In vivo* mitochondrial function remained unchanged upon dapagliflozin treatment (0.008 (−1.745–1.760) s, p = 0.88 Figure 1A). Consistently, *ex vivo* mitochondrial respiratory capacity of permeabilized skeletal muscle fibers obtained after an overnight fast also remained unchanged by dapagliflozin treatment (Figure 1B). Citrate synthase activity, which reflects mitochondrial content, was not significantly affected by dapagliflozin treatment (−0.37 (−1.22, 0.48) μmol∗min^−1^∗gr^−1^, Figure 1C, p = 0.37). Mitochondrial network integrity, as determined by the mitochondrial fragmentation index (MFI) [40], was unaffected upon dapagliflozin treatment irrespective of muscle fiber type (All fibers: 0.13 (−0.12–0.38), p = 0.44; Type I: 0.12 (−0.13–0.37), p = 0.44; Type II: 0.13 (−0.12–0.39), p = 0.44, Suppl. Figure 2A).

**Figure 2:**
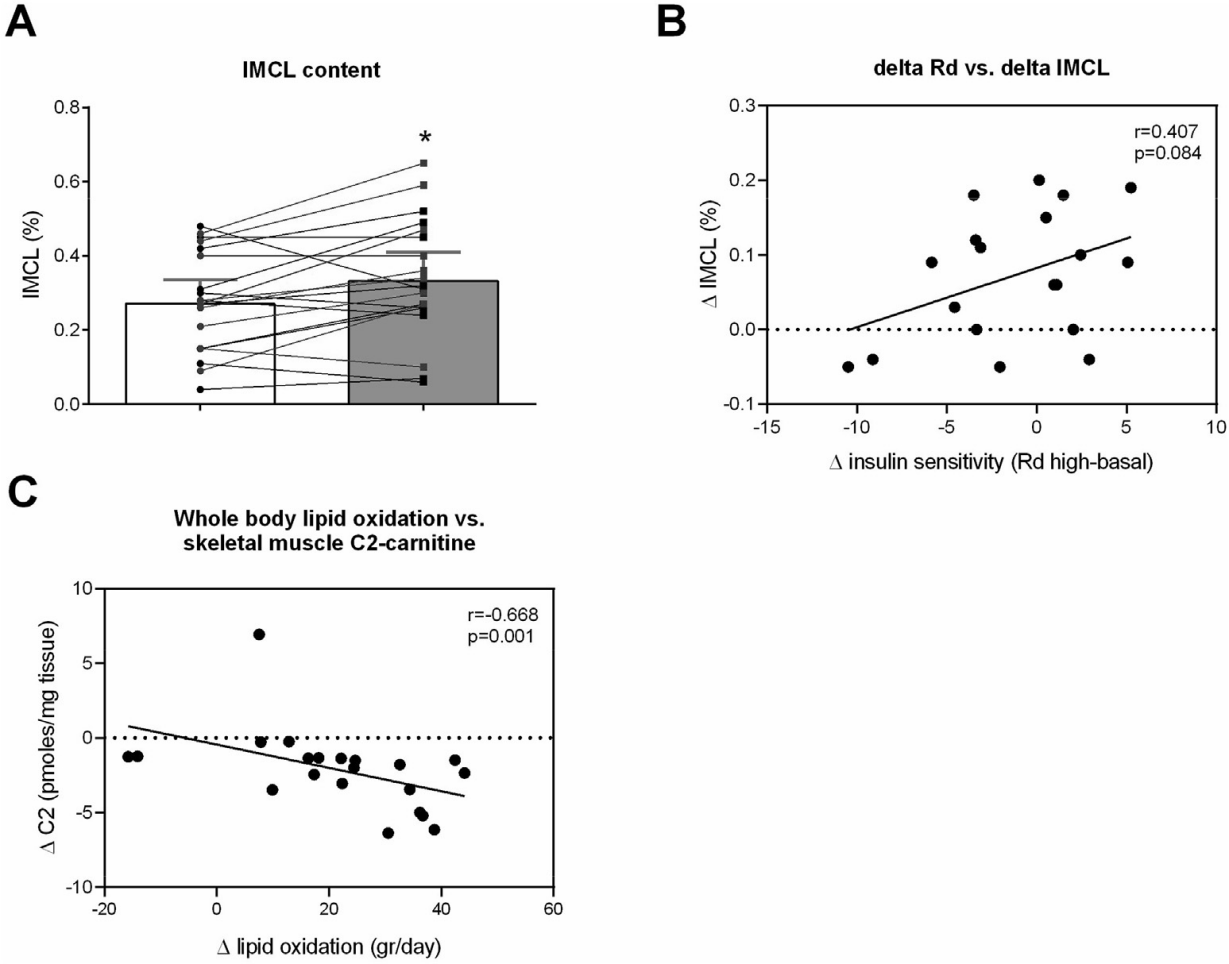
**Increased intramyocellular lipid content after dapagliflozin treatment.(A)** intramyocellular lipid (IMCL) content of m. tibialis anterior measured with ^1^H-MRS and expressed as CH_2_ intensity relative to water resonance (%), **(B)** Pearson correlation between change in insulin sensitivity (delta RD_high-basal_) and change in IMCL, and **(C)** spearman correlation between change in whole-body lipid oxidation and change in skeletal muscle acetylcarnitine (C2 carnitine) levels. Placebo condition = white bars, dapagliflozin condition = grey bars. Results (n = 20) are given as least squares mean (LSM) and 95% confidence interval (CI), obtained through a linear mixed model. ∗P < 0.05 vs. placebo by Wilcoxon paired signed-rank test.

### 3.3 Unaltered skeletal muscle acetylcarnitine levels after dapagliflozin treatment

We previously reported that 5 weeks of dapagliflozin treatment increased 24 h FFA levels and ketone bodies, and reduced 24 h glucose levels, but did not alter whole-body or peripheral insulin sensitivity [6]. The capacity of skeletal muscle to interconvert acetyl-CoA and acetylcarnitine reflects a carbon buffering mechanism that is thought to protect mitochondria against substrate overload [18]. Therefore, we determined *in vivo* acetylcarnitine levels in the postprandial state in the afternoon using ^1^H-MRS before and after 30 min of exercise. Acetylcarnitine levels both prior (0.034 (−0.204–0.272) mmol/kg ww, p = 0.69; Figure 1D) to and immediately after exercise as well as the maximum acetylcarnitine concentration after exercise (0.196 (−0.421–0.813) mmol/kg ww, p = 0.48 and 0.082 (−0.460–0.625) mmol/kg ww, p = 0.66; Figure 1C) remained unchanged with dapagliflozin treatment. During the 30 min of exercise, the increase in plasma lactate levels was lower after dapagliflozin treatment (−0.604 (−1.204, −0.004) mmol/L, p < 0.05, Figure 1E) suggesting reduced glycolysis and glucose flux in skeletal muscle during exercise.

### 3.4 Increased intramyocellular lipid content after dapagliflozin treatment

We previously reported that dapagliflozin decreased trunk fat mass and hepatic lipid content [6] and hypothesized that IMCL content may be reduced as well. Interestingly, IMCL content as measured by ^1^H-MRS in the tibialis anterior muscle was significantly increased after dapagliflozin treatment (0.060 (0.011, 0.110) %, p = 0.019, Figure 2A). From 20 examined participants, 13 displayed an increase in IMCL, 2 had similar and 5 had reduced IMCL content, after dapagliflozin treatment as compared to placebo. Although dapagliflozin did not significantly change peripheral insulin sensitivity on a group level [6], the difference in peripheral insulin sensitivity between the treatment periods (placebo vs. dapagliflozin) tended to correlate positively with the difference in IMCL between the treatment periods (r = 0.407, p = 0.08; Figure 2B) suggesting that the increase in IMCL following dapagliflozin treatment was associated with an improvement in peripheral insulin sensitivity.

### 3.5 Intramyocellular lipid droplet morphology is affected by dapagliflozin treatment, while mitochondrial contact sites are unaffected

**Figure 3:**
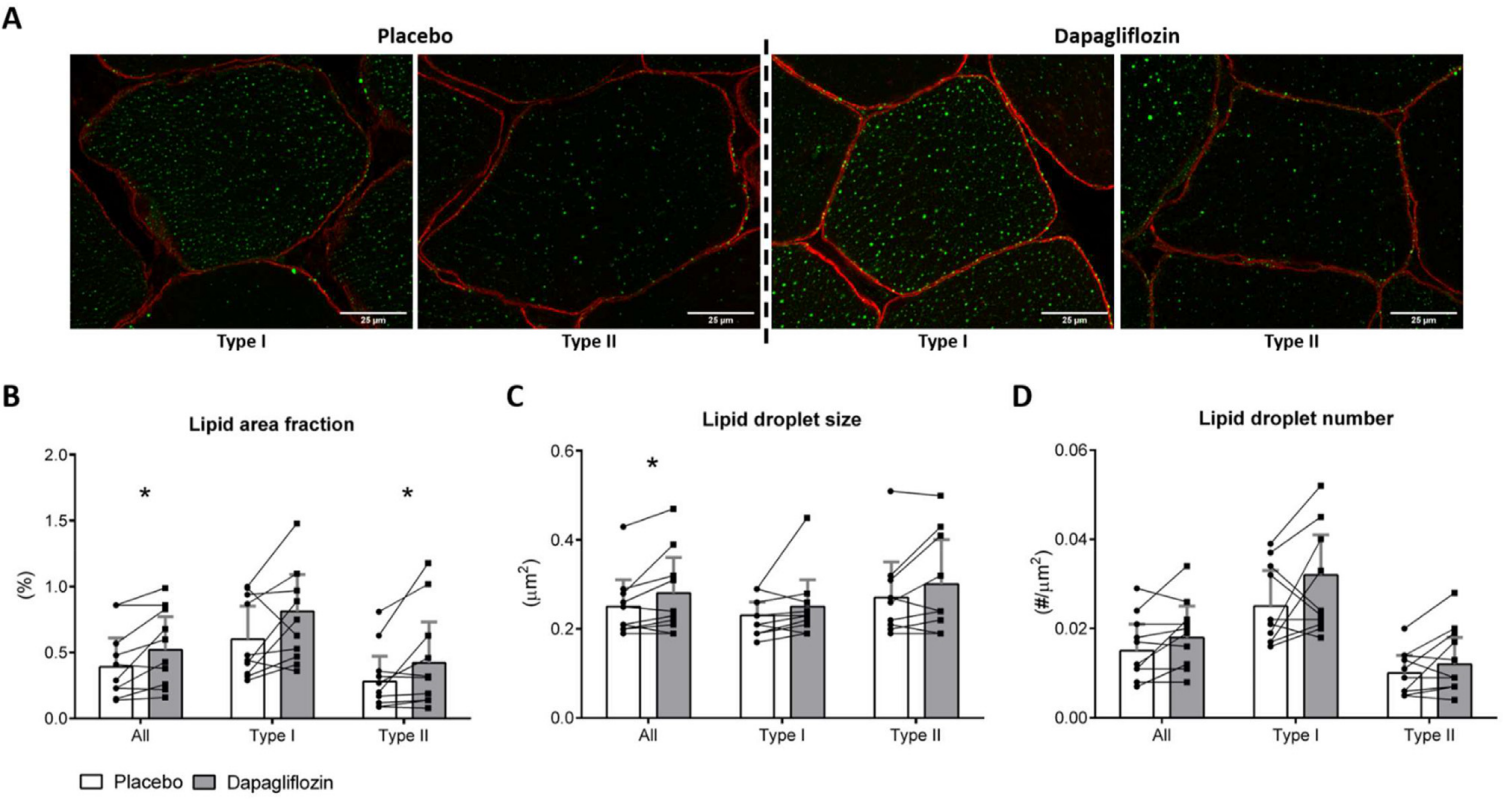
**Effects of dapagliflozin on Intramyocellular lipid droplet morphology in vastus lateralis.(A)** representative images of LDs stained in green and cell membranes stained in red of type 1 and type 2 muscle fibers after placebo and dapagliflozin treatment. **(B)**–**(D)** quantification of lipid area fraction, LD number and LD size respectively. Placebo condition = white bars, dapagliflozin condition = grey bars. Results (n = 10) are in least squares mean (LSM) and 95% confidence interval (CI), obtained through a linear mixed model. ∗P < 0.05 vs. placebo by Wilcoxon paired signed-rank test. Muscle biopsies were taken in the overnight fasted state.

To further examine whether the dapagliflozin-induced increase in IMCL content was due to LD remodeling, i.e. changes in LD number and size and/or fiber type specific effects, we first analyzed lipid fraction area in sections from the vastus lateralis muscle biopsy and then applied confocal microscopy in 10 out of 15 volunteers that showed an increase in lipid area fraction by dapagliflozin as compared to placebo (suppl. Figure 1). By design, the lipid area fraction significantly increased 1.4-fold (0.135 (0.027–0.244) %, p < 0.05, Figure 3A,B) upon dapagliflozin treatment. This increase in IMCL content was observed in both fiber types (Type I (1.4-fold): 0.209 (0.022–0.396) %, p < 0.05; Type II (1.5-fold): 0.14 (−0.032–0.312) %, p < 0.05, Figure 3A,B). Dapagliflozin treatment significantly increased LD size by 1.1-fold (0.03 (0.01–0.06) μm^2^, p < 0.05, Figure 3A,C) and non-significantly increased LD number (1.4-fold, 0.003 (−0.001–0.007) μm^−2^, p = 0.09, Figure 3A,D). Dapagliflozin treatment non-significantly increased LD number in type II fibers (1.4-fold, 0.003 (−0.001–0.007) μm^−2^, p = 0.11, Figure 3A,D). A complete overview of all data and description on subcellular specific changes in LD morphology can be found in the supplemental materials (Suppl. Figure 3).

Since we observed an increased IMCL content in combination with increased 24 h fat oxidation, we hypothesized that the interaction between LDs and mitochondria increase after dapagliflozin treatment [41]. However, the confocal microscopy analysis did not reveal an increased interaction between LDs and mitochondria (All fibers: −0.94 (−2.62–0.74) %, p = 0.20; Type I: −1.76 (−4.01–0.48) %, p = 0.06; Type II: −0.59 (−2.79–1.62) %, p = 0.51, Suppl. Figure 2B).

### 3.6 Altered acylcarnitine levels in muscle after dapagliflozin treatment

**Figure 4:**
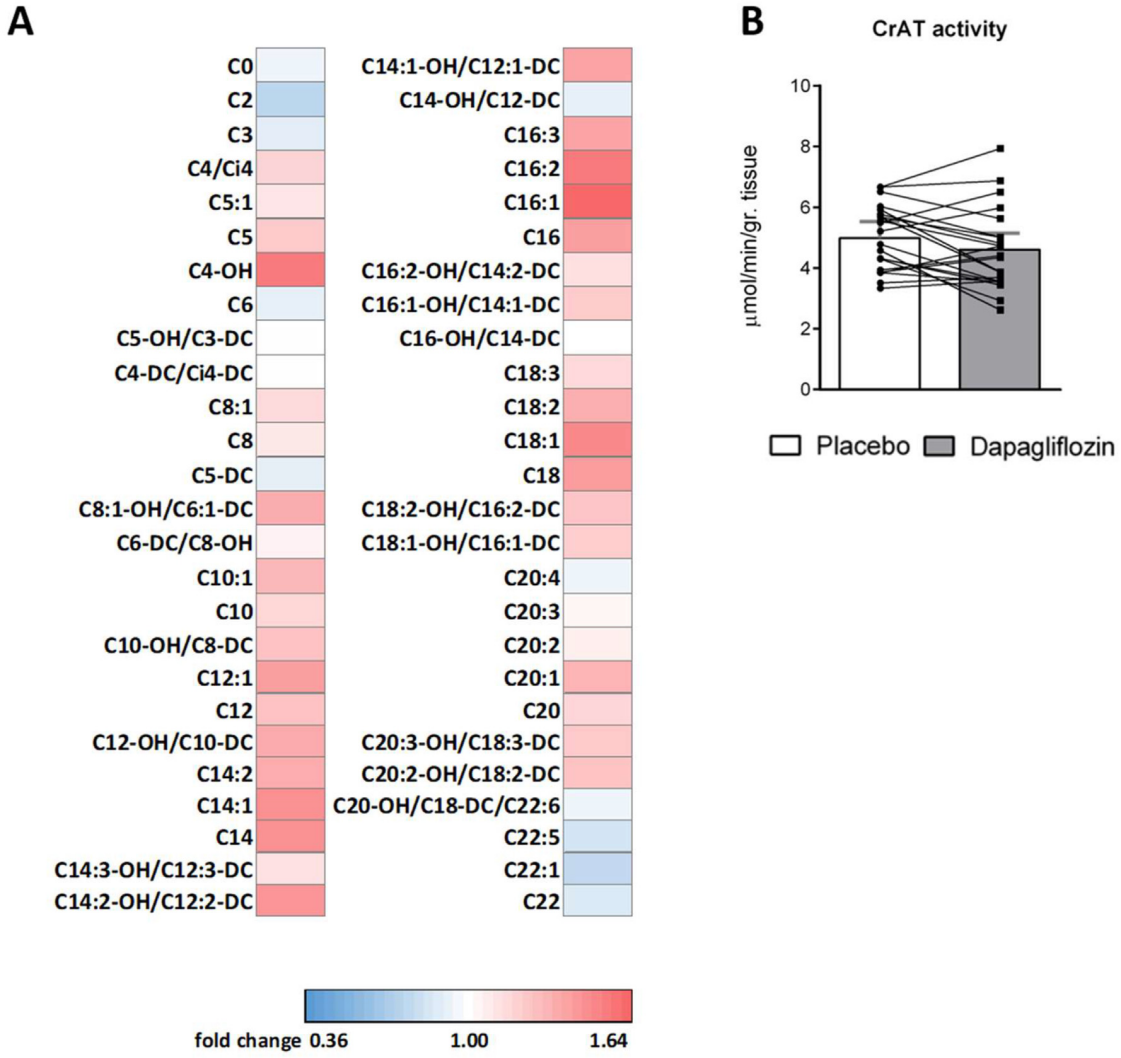
**Effects of dapagliflozin on fasting acylcarnitine and CrAT activity.(A)** Fold change in skeletal muscle levels of carnitine species after an overnight fast (n = 22), and **(B)** creatine acetyltransferase activity (n = 22). Results in b are in least squares mean (LSM) and 95% confidence interval (CI), obtained through a linear mixed model.

To further investigate if the increase in IMCL following dapagliflozin treatment parallels the effects observed with exercise training and/or calorie restriction, we next investigated acylcarnitine species in muscle biopsies as a marker of muscle-specific fatty acid metabolism. In contrast to the determination of acetylcarnitine by MRS in the afternoon in the postprandial state, when muscle relies less on fatty acid oxidation, acetylcarnitine (C2) levels, measured in muscle biopsies taken after an overnight fast, were decreased after dapagliflozin treatment (−40.0774 (−64.4766, −15.6782) pmoles∗mg tissue^−1^, p < 0.001, Figure 4A). The decrease in acetylcarnitine levels by dapagliflozin correlated with the increase in 24 h fat oxidation (r = −0.668, p < 0.01; Figure 2C). Carnitine acetyltransferase (CrAT) activity was non-significantly reduced after dapagliflozin treatment (−0.381 (−0.796, 0.033), p = 0.069, Figure 4B). However, all other acylcarnitines metabolites were generally higher after dapagliflozin treatment, which together with elevated IMCL levels is suggestive of an increased supply and flux of fatty acids in skeletal muscle. Thus, levels of C4–OH (0.4704 (0.1246, 0.8162) pmoles∗mg tissue^−1^, p < 0.001, Figure 4A) were significantly higher after dapagliflozin compared to placebo. Similarly, several long-chain acylcarnitines were also significantly higher after dapagliflozin treatment (Figure 4A). A complete overview of all acylcarnitines in skeletal muscle can be found in Figure 4A.

### 3.7 Expression of fatty acid metabolism related genes in skeletal muscle are altered upon dapagliflozin treatment

**Figure 5:**
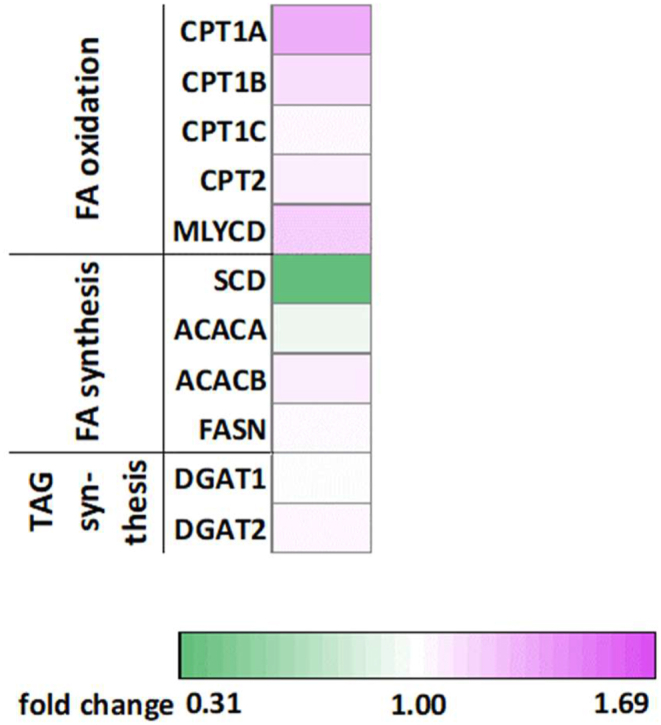
**Effects of dapagliflozin on expression of genes involved in fatty acid metabolism.** Fold change in expression of genes in skeletal muscle fatty acid metabolism (n = 22).

The results from the whole-body calorimetry and increased FFA plasma levels [6] and muscle levels of acylcarnitines indicate an increase in skeletal muscle fatty acid oxidation, while total IMCL levels were increased after dapagliflozin treatment. Expression of genes involved in fatty acid metabolism was therefore investigated to get a better understanding of metabolic adjustments that could help to explain these results. Genes regulating fatty acid mitochondrial import, such as CPT1A (1.33-fold), CPT1B (1.12-fold) and MLYCD (1.19-fold) were significantly upregulated by dapagliflozin treatment. In addition, SCD (−3.24 fold) was significantly decreased after dapagliflozin treatment. An overview of measured genes related to lipid metabolism can be found in Figure 5.

### 3.8 Lower amino acids and TCA cycle intermediates in skeletal muscle after dapagliflozin treatment

**Figure 6:**
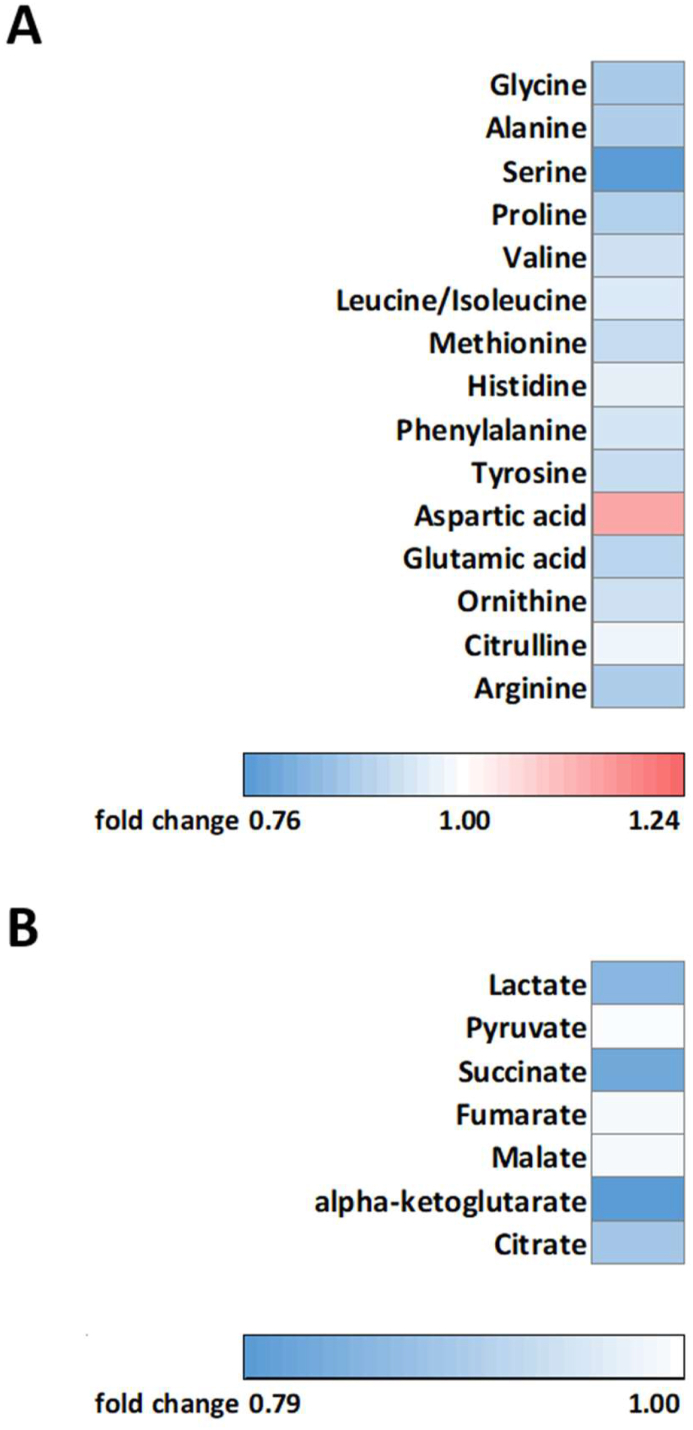
**Amino acids and TCA cycle intermediates levels.(A)** Fold change in skeletal muscle levels of amino acids after an overnight fast (n = 22), and **(B)** fold change in skeletal muscle levels of TCA cycle intermediates (n = 22).

To further investigate if dapagliflozin affected skeletal muscle substrate metabolism, we also measured levels of amino acids and TCA cycle intermediates in the muscle biopsies. We found that levels of alanine (−150.16 (−245.32, −55.0091) pmoles∗mg tissue^−1^, p = 0.013), proline (−46.0350 (−78.7122, −13.3578) pmoles∗mg tissue^−1^, p = 0.0094), valine (−14.6018 (−26.1708, −3.0327) pmoles∗mg tissue^−1^, p = 0.0094), and glutamic acid (−166.71 (−299.82, −33.6025) pmoles∗mg tissue^−1^, p = 0.015) were lower after dapagliflozin treatment compared to placebo (Figure 6A). Furthermore, and consistent with the lower plasma lactate levels during exercise, skeletal muscle lactate levels were lower after dapagliflozin treatment in the fasted state (−464.01 (−840.41, −87.6004) pmoles∗mg tissue^−1^, p = 0.033, Figure 6B). Furthermore, the TCA cycle intermediates succinate (−15.26 (−27.37, −3.15) pmoles∗mg tissue^−1^, p = 0.007, Figure 6B) and alpha-ketoglutarate (−5.51 (−11.45, −0.42) pmoles∗mg tissue^−1^, p = 0.013, Figure 6B) was significantly lower after dapagliflozin treatment, while citrate (−14.4604 (−28.4890, −0.4317) pmoles∗mg tissue^−1^, p = 0.060, Figure 6B) showed a trend towards lower levels after dapagliflozin treatment. Together, these findings indicate a reduced glycolysis and glucose oxidation in skeletal muscle. In addition, the reduced amino acid levels may be explained by mobilization of muscle-derived amino acids for hepatic gluconeogenesis. An overview of all amino acids and TCA cycle intermediates can be found in Figure 6.

## 4 Discussion

We previously reported that 5-weeks of dapagliflozin treatment in patients with type 2 diabetes increased whole-body 24 h fat oxidation, reduced hepatic lipid content, trunk fat mass, weight (−1.26 kg) and lean mass (−0.67 kg), while total energy expenditure did not change [6]. In addition, we did not observe a change in whole-body or peripheral insulin sensitivity despite an increase in plasma FFA levels and whole-body fatty acid oxidation [6]. Since the dapagliflozin-induced loss of glucose in the urine can be regarded as a form of mild calorie restriction, we here investigated the hypothesis that SGLT2 inhibition induces calorie restriction-like effects at the cellular level of the skeletal muscle, including changes in skeletal muscle fatty acid metabolism. We show that dapagliflozin had pronounced effects on skeletal muscle cellular metabolism including alterations in the expression of fatty acid handling genes, levels of amino acids and TCA cycle intermediates, acylcarnitines and increased size and number of lipid droplets, but dapagliflozin did not affect skeletal muscle mitochondrial function and mitochondrial network integrity.

SGLT2 is mainly present in the kidney and with a very lowly expression in the intestines [4]. SGLT2 inhibition induces glucose lowering effects due to a direct drain of glucose – and therefore calories – into the urine; therefore, any effects observed on skeletal muscle metabolism is a secondary effect of either the lowering of plasma glucose or the loss of calories. Although we cannot exclude that the effects observed are due to the lowering of plasma glucose, it should be noted that SGTL2 inhibitors are the only glucose-lowering antidiabetic drugs that are accompanied by increases in plasma FFA levels and elevated fat oxidation. Given that these effects are also markers of calorie restriction, the latter is a more logical explanation for the effects observed on skeletal muscle.

Dapagliflozin treatment had marked effects on skeletal muscle acylcarnitine species, including elevated long-chain acylcarnitines and lower acetylcarnitine levels in the overnight, fasted state. It has been shown that 12 h fasting in obese participants decreased acetylcarnitine levels [42]. Together with higher long-chain acylcarnitines levels, this may reflect diminished glucose uptake and oxidation by skeletal muscle coupled with increased influx and oxidation of fatty acids. Consistently, we found that the change in acetylcarnitine levels between treatments correlated negatively with the change in whole-body fat oxidation. In addition to the elevated acylcarnitine levels in muscle, skeletal muscle C4–OH was also increased. C4–OH has been linked to both fasting and ketosis, and can be derived from the ketone body D-(−)-3-hydroxybutyrate (D-3HB) [43] as well as from long chain fatty acids, and may reflect enhanced utilization of both ketone bodies and fatty acids. In addition, expression of CPT1a and CPT1b involved in mitochondrial transport of fatty acids were increased, while the genes related to triglyceride synthesis were unaffected. Together, our results suggest that dapagliflozin reduces glucose use while increasing ketone and fatty acid utilization in muscle cells, effects similar to those observed after calorie restriction [44] and/or prolonged exercise training [45].

We next investigated if the increased plasma FFA [6] levels also affected intramyocellular lipid stores. Interestingly, IMCL increased after dapagliflozin treatment, which seems paradoxical when considering the changes indicating increased skeletal muscle fatty acid oxidation and no change in peripheral insulin sensitivity [6]. However, we and others have previously shown that interventions that raise circulating FFA levels leading to increased skeletal muscle fatty acid oxidation, such as exercise training, resveratrol treatment and prolonged fasting, also increase the level of IMCL [12,46,47]. Under such conditions, increased IMCL is not necessarily associated with deteriorated insulin sensitivity, but merely reflects an enhanced storage capacity accompanying the enhanced reliance of fatty acids for oxidation. We have recently shown that IMCL storage under healthy trained conditions mainly involved the storage of lipids in small LDs in type I muscle fibers, whereas in diabetes patients, skeletal muscle lipids are mainly stored as large LDs in type II muscle fibers [21]. Here, we show that the increased IMCL after dapagliflozin was due to both an increase in LD number as well as larger LDs. We have observed previously that an increase in IMCL content due to both LD number and size occurs with interventions increasing muscle fatty acid oxidation such as prolonged fasting [22] and resveratrol [48]. The increased IMCL storage capacity and LD size may therefore be reflective of altered LD dynamics similar to what is seen with fasting. Thus, the small increase in LD size without an alteration in insulin sensitivity suggests that upon dapagliflozin treatment LDs grow in size by incorporating free fatty acids into triglycerides that are not needed for oxidation, and thereby matches free fatty acid flux to mitochondrial oxidation rates. In such a model, lipotoxicity is prevented, and may ultimately even improve insulin sensitivity, as has been reported for SGTL2i treatment by others [26,27].

In addition to increased fatty acid oxidation, we here report lower levels of TCA cycle intermediates and lactate in skeletal muscle. Such changes in TCA cycle intermediates may be reflective of a lower glycolytic flux [42] and the shuttling of glucogenic skeletal muscle amino acids (valine, proline, alanine and glutamic acid) to the liver for gluconeogenesis and urea production [49]. Indeed, valine, proline, alanine and glutamic acid decreased after dapagliflozin treatment and urea levels were previously reported to increase by dapagliflozin [6]. Specifically glucogenic amino acids in skeletal muscle are reported to decrease after fasting or starvation, and are thought to indicate an increased efflux to plasma in order to act as a substrate for hepatic gluconeogenesis [50]. We [6] and others [27] have shown that SGLT2i increases endogenous glucose production in the fasting state. The current findings therefore may suggest enhanced flux of amino acids from skeletal muscle for gluconeogenesis. In addition, transamination of amino acids in liver can be used for the biosynthesis of urea, which may help to maintain the osmotic pressure in kidney medulla to prevent excessive water loss following SGTL2 inhibition [49]. Of note, a switch in skeletal muscle energy use away from glycolytic substrate towards energy generation from fatty acids, also reduces the need for muscle protein breakdown. Consistently, no sign of muscle protein breakdown or elevated urine nitrogen excretion was observed [6]. We have suggested that the increase in EGP upon dapagliflozin in our study may be driven by increased delivery of both fatty acids and glycerol, in which the latter also provides a carbon precursor for hepatic gluconeogenesis thereby limiting the use of amino acids for gluconeogenesis [6]. Consistently, also after prolonged fasting or starvation there is a consistent contribution of glycerol to gluconeogenesis [51].

Maximal mitochondrial respiratory capacity was unaltered after SGLT2 inhibition. In order to maintain properly functioning mitochondria, the mitochondrial network undergoes a continuous cycling of fission and fusion [52]. In line with the unaltered mitochondrial function, mitochondrial network integrity was unaltered upon dapagliflozin treatment. Consistently, calorie restriction in overweight to obese participants did not alter mitochondrial function or density [53]. Moreover, we previously showed that prolonged fasting for 60 h in healthy lean participants – if anything – slightly reduced mitochondrial function [54]. These finding suggest that shifts in mitochondrial fatty acid oxidation and/or fatty acid flux are not necessarily caused by changes in (maximal) mitochondrial respiration. Increased interaction between LDs and mitochondria could also help to explain a more metabolically healthy skeletal muscle phenotype [21]. However, the number of mitochondrial contact sites at the lipid droplet surface was not changed by dapagliflozin.

## 5 Conclusions

To summarize, dapagliflozin treatment for 5 weeks resulted in changes in skeletal muscle cellular metabolism resembling more the state of fasting than alterations induced by exercise training and favoring the metabolism of fatty acid and ketone bodies and moving away from glycolytic flux. The long-term effects of such muscle adaptions for cardiovascular and metabolic health, as well as the investigation of similar effects on other metabolic tissues such as the heart, needs future research.

## Funding

The study was funded by AstraZeneca. The study funder was involved in the design of the study, the interpretation of data, and writing the report, and did not impose any restrictions regarding the publication of the report.

## Author contribution

P.S., V.S.H., M.H., J.H., E.P., B.H., J.O. and R.E. designed and conceived the study. Y.K., M.L., B.D. E.P., E.K., G.S., J.J., J.H., M.H., V.S.H., T.K., D.M., E.P. and P.S. designed and performed the experiments. Y.K., A.G., M.L., B.D., D.P., V.S.H., T.K., D.M., E.P., and P.S. analyzed the data. Y.K., A.G., P.S. and J.O. drafted the manuscript. All authors reviewed and approved the final version of the manuscript. P.S is the guarantor of this work and, as such, had full access to all the data in the study and takes responsibility for the integrity of the data and the accuracy of the data analysis.

## Data sharing

Data underlying the findings described in this manuscript may be available upon request in accordance with AstraZeneca's data sharing policy described at https://astrazenecagroup-dt.pharmacm.com/DT/Home.

## Data Availability

Data described in this manuscript may be available upon request in accordance with AstraZeneca's data sharing policy described at https://astrazenecagroup-dt.pharmacm.com/DT/Home
