# Supplementary material for: Effects of SGLT2 inhibitor dapagliflozin in patients with type 2 diabetes on skeletal muscle cellular metabolism
Source: Mol Metab. 2022 Oct 21;66:101620. doi: 10.1016/j.molmet.2022.101620 (PMC9636471; doi:10.1016/j.molmet.2022.101620)
Supplement: Multimedia component 1 [file mmc1.docx]

**SUPPLEMENTAL MATERIALS**

**METHODS**

**Procedures**

In short, the study comprised seven visits, including the screening (Visit 1). Visit 2 included randomisation of the participants. This visit and corresponding visit during the next period (Visit 5) safety markers were measured. After two weeks of treatment, a safety visit (Visit 3 or 6) followed, and an end-of-treatment visit (Visit 4 or 7) after approximately 5 weeks of treatment. All outcome measures were performed at Visit 4 and 7 and were spread over a period of 6-8 days. Visit 4 was followed by a 6-8 weeks wash-out period. The primary outcome parameter was comparison of dapagliflozin versus placebo after 5 weeks treatment on skeletal muscle insulin sensitivity measured as the change in glucose disposal rate from basal to the high-insulin state and corrected for urinary glucose loss using a 2-step euglycemic hyperinsulinemic clamp procedure (1). Exploratory endpoints were whole-body 24h energy expenditure and substrate oxidation, whole-body and tissue-specific insulin sensitivity and body composition including liver fat accumulation which have been published previously (1).

**Maximal VO2 cycling test**

For baseline characterization (Visit 2), participants performed a graded cycling test to determine maximal oxygen uptake (VO2max) and maximal power output (Wmax) as previously described (2) for the measurements of acetylcarnitine before and after exercise with 1H magnetic resonance spectroscopy (1H-MRS).

**Maximal knee-extension test**

A maximal knee extension test was performed at Visit 2 to determine exercise intensity of 60% of maximal weight to be used during the phosphocreatine (PCr) recovery 31P-MRS measurements scheduled for Visits 4 and 7. This test was performed on a MR- compatible knee-extension exercise device, with incremental weight put on the left leg (500 g every 30 seconds) until exhaustion (3).

**Magnetic resonance spectroscopy measurements**

*1H-MRS for in vivo IMCL content*

The ^1^H-MRS spectra obtained were analysed and fitted in the time domain by using the non-linear least-squares Advanced Method for Accurate, Robust, and Efficient Spectral (AMARES) algorithm (4, 5), in the java-based magnetic resonance user interface (jMRUI) software package (6) as described earlier (7). IMCL is given as percentage of the CH_2_ peak compared with the water resonance and corrected for T1 and T2 relaxation time. IMCL could not be measured in one participant due to technical failure.

*31P-MRS for PCr recovery rate*

A knee-extension protocol was performed for 5 minutes, on a custom-built magnetic resonance-compatible ergometer with a pulley system in a 3.0 T whole-body MRI scanner (Achieva 3Tx; Philips Healthcare). A coil (5 cm diameter) was positioned on the vastus lateralis muscle and a time series of ^31^P-MRS spectra (free induction decays) were acquired during 12 minutes (2 minutes of rest, 5 minutes of exercise and 2 minutes recovery) with a repetition time of 4 seconds. Post-exercise PCr kinetics was computed as previously described and given as half-time of PCr recovery (s) (3).

*1H-MRS for acetylcarnitine concentrations during and after exercise*

Resting skeletal muscle acetylcarnitine concentrations were measured at 5 P.M. using a T1-editing method, as described previously (8), after which volunteers performed a 30 min cycling exercise at 70% maximal output on an ergometer. Immediately following exercise, acetylcarnitine concentrations were measured again. Acetylcarnitine values were converted to absolute concentrations as described previously (9). The creatine peak was used as a reference. During the 30 minutes cycling, blood draws were performed to measure lactate levels at the start, after 15 minutes and directly after cycling (30 minutes).

**Confocal microscopy analyses**

*Staining procedures*

Five µm thick sections were made and were mounted on glass slides. To minimize variability in staining intensities between the placebo and dapagliflozin treatment arms, sections of both treatment arms of the same participant was mounted on the same glass slide. Sections were fixated with 3.7% formaldehyde for 30 minutes and subsequently stained with antibodies against TOMM20 (Ab186734, Abcam, Cambridge, UK), caveolin (610421, BD Biosciences, Franklin Lakes, NJ, USA) and myosin heavy chain type I (A4.840, Developmental Studies Hybridoma Bank, Iowa City, IA, USA). Lipid droplets (LDs) were visualized with Bodipy 493/503. Subsequently, a combination of appropriately conjugated secondary antibodies AlexaFluor405, AlexFluor555 and AlexaFluor647 was used. Sections were mounted with Mowiol, covered with #1 coverslips and stored in the dark until imaging.

*Image analysis*

Image analysis pipelines for LD morphology and location (10), and mitochondrial network integrity (11) have been described previously. Z-stacks acquired for the analysis of LD-mitochondrial interaction were filtered using a difference of Gaussian filter for the LDs and for the mitochondria a white top hat filter. In order to quantify mitochondrial contact sites at the LD surface binary images were created from these filtered images. The Label Boundaries tool of the MorphoLibJ plugin (12) was used to isolate the LD surface. Subsequently, binary images of mitochondrial contact sites on the LDs were created using the image calculator in ImageJ with the LD surface and mitochondria images as input and using the AND operater. From the images containing the LD surface and mitochondrial contact sites the percentage of the LD surface in touch with mitochondria were determined.

**RNA sequencing**

RNA concentration was measured with a Nanodrop 1000 spectrometer and RNA integrity was determined using an Agilent 2100 Bioanalyzer with RNA 6000 microchips (Agilent Technologies, Santa Clara, CA). Library construction and RNA sequencing runs on the BGISEQ-500 platform (13) were conducted at Beijing Genomics Institute (BGI, Denmark). At BGI, Genomic DNA was removed with two digestions using Amplification grade DNAse I (Invitrogen, USA). The RNA was sheared and reverse transcribed using random primers to obtain cDNA, which was used for library construction. The library quality was determined by using Bioanalyzer 2100. Then, the library was used for 100bp paired-end sequencing on the sequencing platform BGISEQ-500 (BGI).

**Processing of RNA sequencing reads**

The RNA-seq reads were used to quantify transcript abundances. To this end the tool *Salmon* (14) (version 1.5.2) was used to map the reads to the GRCh38.p13 human genome assembly-based transcriptome sequences as annotated by the GENCODE consortium (15) (release 38). The obtained transcript abundance estimates and lengths were then imported in R using the package *tximport* (16) (version 1.22.0), scaled by average transcript length and library size, and summarized on the gene-level. Such scaling corrects for bias due to correlation across samples and transcript length, and has been reported to improve the accuracy of differential gene expression analysis (16). Differential gene expression was determined using the package *limma* (17) (version 3.50.0) utilizing the obtained scaled gene-level counts. Briefly, before statistical analyses, nonspecific filtering of the count table was performed to increase detection power (18), based on the requirement that a gene should have an expression level greater than 10 counts, i.e. ~0.5 count per million reads (cpm) mapped, for at least 16 libraries across all 45 samples. Differences in library size were adjusted by the trimmed mean of M-values normalization method (19), implemented in the package *edgeR* (20) (version3.36.0). Only protein-coding genes were retained, whereafter counts were transformed to log2(cpm) values and associated precision weights, and entered into the *limma* analysis pipeline (21). Differentially expressed genes were identified by using generalized linear models that incorporate empirical Bayes methods to shrink the standard errors towards a common value, thereby improving testing power (17, 22). Genes were defined as significantly changed when P < 0.05.

**RESULTS**

**Changes in lipid droplet morphology in the IMF and SS subcellular regions upon dapagliflozin treatment**

The increase in IMCL content observed when taken all fibers together were observed in both the intermyofibrillar (IMF) and subsarcolemmal (SS) regions (IMF (1.5-fold): 0.125 (0.039-0.212) %, p<0.05; SS (1.4-fold): 0.287 (-0.073-0.647) %, p=0.09, Suppl. Figure 3a). It was observed that dapagliflozin treatment had differential effects on LD morphology in relation to fiber types and subcellular localization. A trend towards larger LDs following dapagliflozin treatment was observed both in the IMF (1.1 fold) (0.03 (0.00-0.05) µm^2^, p=0.08, Suppl. Figure 3a) and SS (1.2-fold, 0.05 (0.00-0.10) µm^2^, p=0.05, Suppl. Figure 3b) region. This trend towards an increased LD size in the IMF region was more pronounced in the type I fibers (1.1-fold, 0.02 (-0.02-0.05) µm^2^, p=0.10, Suppl. Figure 3a), while the observed larger LDs in the SS region after dapagliflozin treatment was more pronounced in the type II fibers (1.2 fold, 0.06 (0.01-0.10) µm^2^, p<0.05, Suppl. Figure 3b). The increased in LD number in the type II fibers was significant for the IMF region (1.6 fold, 0.003 (0.000-0.007) µm^-2^, p<0.05, Suppl. Figure 3a)

*Supplemental Table 1. Effects of dapagliflozin treatment on glycaemic control. Results are presented as least square means (95% CI) or mean±SD.*

| **Parameter** | **Placebo** | **Dapagliflozin** | **p-value** |
| --- | --- | --- | --- |
| Fasting glucose (mmol/L) | 8.89±1.92 | 7.82±1.39 | p<0.001 |
| Fasting insulin (pmol/L) | 61.29 (52.90-69.68) | 43.12 (34.73-51.51) | p<0.001 |
| HbA_1C_ (%) | 6.96 (6.62-7.30) | 6.89 (6.55-7.23) | p=0.33 |
| Fasting NEFA (μmol/L) | 590.9 (455.3-726.6) | 799.8 (667.8-931.9) | p<0.05 |
| Fasting glycerol (mmol/L) | 0.046 (0.032-0.059) | 0.067 (0.054-0.081) | p<0.05 |
| 24-h glucagon AUC (pmol/L/h) | 300.1 (248.4-351.8) | 325.0 (273.3-376.7) | p<0.05 |
| hsCRP (mg/L) | 1.17 (0.52-1.82) | 1.39 (0.74-2.04) | p=0.50 |
| Urate (μmol/L) | 324.5 (298.2-350.7) | 267.7 (241.4-294.0) | p<0.001 |





**Supplemental Figure 1. Participant selection for detailed lipid droplet morphology and mitochondrial network integrity analysis.** Based on analysis of vastus lateralis skeletal muscle biopsies stained with Bodipy 493/503 for IMCL content with widefield microscopy (p=0.378, Wilcoxon Signed Ranked Test), 10 participants out of 15 participants showing an increase in IMCL content were selected for a detailed analysis for LD morphology and location, and mitochondrial network integrity with confocal microscopy. Selected participants are shown with a green line. 8 out of 23 participants showed a decrease. Placebo condition = white bars, dapagliflozin condition = grey bars. Results (n=23) are in least squares mean (LSM) and 95% confidence interval (CI), obtained through a linear mixed model. * P<0.05 vs. placebo by Wilcoxon paired signed-rank test.

**Supplemental Figure 2. Unaltered mitochondrial network integrity after dapagliflozin treatment.** a) representative images of the mitochondrial networks of type 1 and type 2 fibers after placebo and dapagliflozin treatment, and quantification of the mitochondrial network integrity. b) quantification of percentage surface of the lipid droplets covered by mitochondria. Placebo condition = white bars, dapagliflozin condition = grey bars. Results (n=9 for MFI and n=10 for LD-mitochondrial contact sites) are in least squares mean (LSM) and 95% confidence interval (CI), obtained through a linear mixed model. * P<0.05 vs. placebo by Wilcoxon paired signed-rank test.


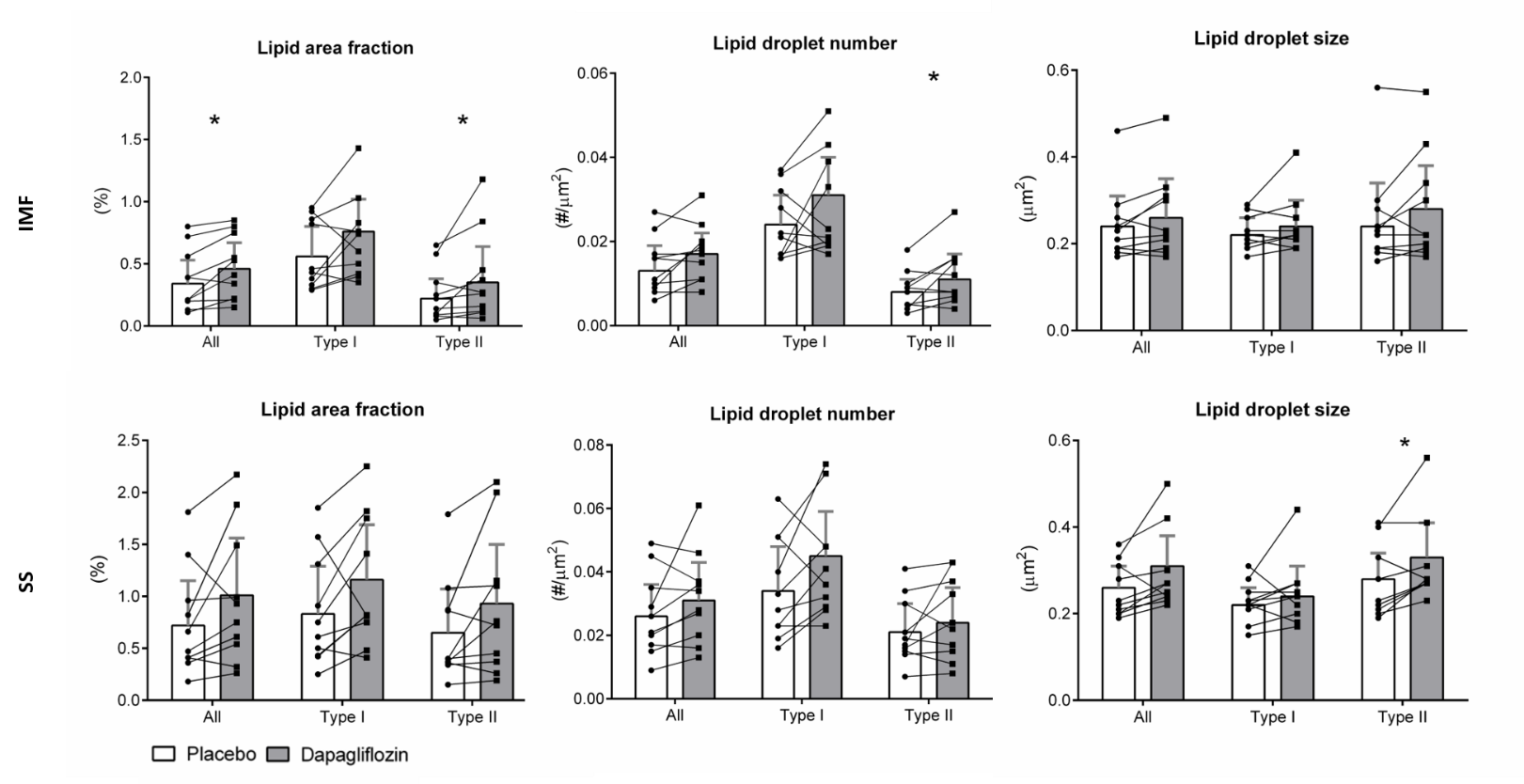


**Supplemental Figure 3. Changes in lipid droplet characteristics in the intermyofibrillar and subcellular region.** Quantification of lipid area fraction, LD number and LD size in the a) IMF and b) SS regions for each fiber type after placebo and dapagliflozin treatment. Placebo condition = white bars, dapagliflozin condition = grey bars. Results (n=10) are in least squares mean (LSM) and 95% confidence interval (CI), obtained through a linear mixed model. * P<0.05 vs. placebo by Wilcoxon paired signed-rank test.

**REFERENCES**

1. Op den Kamp YJM, de Ligt M, Dautzenberg B, Kornips E, Esterline R, Hesselink MKC, et al. Effects of the SGLT2 Inhibitor Dapagliflozin on Energy Metabolism in Patients With Type 2 Diabetes: A Randomized, Double-Blind Crossover Trial. Diabetes Care. 2021;44(6):1334-43.

2. Kuipers H, Verstappen FT, Keizer HA, Geurten P, van Kranenburg G. Variability of aerobic performance in the laboratory and its physiologic correlates. International journal of sports medicine. 1985;6(4):197-201.

3. Schrauwen-Hinderling VB, Kooi ME, Hesselink MK, Jeneson JA, Backes WH, van Echteld CJ, et al. Impaired in vivo mitochondrial function but similar intramyocellular lipid content in patients with type 2 diabetes mellitus and BMI-matched control subjects. Diabetologia. 2007;50(1):113-20.

4. Krssak M, Mlynarik V, Meyerspeer M, Moser E, Roden M. 1H NMR relaxation times of skeletal muscle metabolites at 3 T. MAGMA. 2004;16(4):155-9.

5. Vanhamme L, van den Boogaart A, Van Huffel S. Improved method for accurate and efficient quantification of MRS data with use of prior knowledge. J Magn Reson. 1997;129(1):35-43.

6. Naressi A, Couturier C, Devos JM, Janssen M, Mangeat C, de Beer R, et al. Java-based graphical user interface for the MRUI quantitation package. MAGMA. 2001;12(2-3):141-52.

7. Lindeboom L, Nabuurs CI, Hesselink MK, Wildberger JE, Schrauwen P, Schrauwen-Hinderling VB. Proton magnetic resonance spectroscopy reveals increased hepatic lipid content after a single high-fat meal with no additional modulation by added protein. Am J Clin Nutr. 2015;101(1):65-71.

8. Lindeboom L, Bruls YM, van Ewijk PA, Hesselink MK, Wildberger JE, Schrauwen P, et al. Longitudinal relaxation time editing for acetylcarnitine detection with (1) H-MRS. Magn Reson Med. 2017;77(2):505-10.

9. Lindeboom L, Nabuurs CI, Hoeks J, Brouwers B, Phielix E, Kooi ME, et al. Long-echo time MR spectroscopy for skeletal muscle acetylcarnitine detection. J Clin Invest. 2014;124(11):4915-25.

10. Daemen S, Gemmink A, Brouwers B, Meex RCR, Huntjens PR, Schaart G, et al. Distinct lipid droplet characteristics and distribution unmask the apparent contradiction of the athlete's paradox. Mol Metab. 2018;17:71-81.

11. Halling JF, Jessen H, Nohr-Meldgaard J, Buch BT, Christensen NM, Gudiksen A, et al. PGC-1alpha regulates mitochondrial properties beyond biogenesis with aging and exercise training. American journal of physiology Endocrinology and metabolism. 2019;317(3):E513-E25.

12. Legland D, Arganda-Carreras I, Andrey P. MorphoLibJ: integrated library and plugins for mathematical morphology with ImageJ. Bioinformatics. 2016;32(22):3532-4.

13. Goodwin S, McPherson JD, McCombie WR. Coming of age: ten years of next-generation sequencing technologies. Nat Rev Genet. 2016;17(6):333-51.

14. Patro R, Duggal G, Love MI, Irizarry RA, Kingsford C. Salmon provides fast and bias-aware quantification of transcript expression. Nature methods. 2017;14(4):417-9.

15. Frankish A, Diekhans M, Ferreira AM, Johnson R, Jungreis I, Loveland J, et al. GENCODE reference annotation for the human and mouse genomes. Nucleic Acids Res. 2019;47(D1):D766-D73.

16. Soneson C, Love MI, Robinson MD. Differential analyses for RNA-seq: transcript-level estimates improve gene-level inferences. F1000Res. 2015;4:1521.

17. Ritchie ME, Phipson B, Wu D, Hu Y, Law CW, Shi W, et al. limma powers differential expression analyses for RNA-sequencing and microarray studies. Nucleic Acids Res. 2015;43(7):e47.

18. Bourgon R, Gentleman R, Huber W. Independent filtering increases detection power for high-throughput experiments. Proc Natl Acad Sci U S A. 2010;107(21):9546-51.

19. Robinson MD, Oshlack A. A scaling normalization method for differential expression analysis of RNA-seq data. Genome Biol. 2010;11(3):R25.

20. Robinson MD, McCarthy DJ, Smyth GK. edgeR: a Bioconductor package for differential expression analysis of digital gene expression data. Bioinformatics. 2010;26(1):139-40.

21. Law CW, Chen Y, Shi W, Smyth GK. voom: Precision weights unlock linear model analysis tools for RNA-seq read counts. Genome Biol. 2014;15(2):R29.

22. Smyth GK. Linear models and empirical bayes methods for assessing differential expression in microarray experiments. Stat Appl Genet Mol Biol. 2004;3:Article3.
